# What Makes You a Whistleblower? A Multi-Country Field Study on the Determinants of the Intention to Report Wrongdoing

**DOI:** 10.1007/s10551-022-05089-y

**Published:** 2022-03-25

**Authors:** Hengky Latan, Charbel Jose Chiappetta Jabbour, Murad Ali, Ana Beatriz Lopes de Sousa Jabbour, Tan Vo-Thanh

**Affiliations:** 1 HLC Consulting, Jl. Kertanegara Selatan V No 5B, Semarang, 50241 Indonesia; 2 EMLYON Business School, 23 Av. Guy de Collongue, 69130 Écully, France; 3 Affiliate Professor, Lincoln International Business School, University of Lincoln, Brayford Pool, Lincoln, LN6 7TS Lincolnshire UK; 4 Newcastle Business School, Northumbria University, City Campus East 1, Newcastle Upon Tyne, NE1 8ST UK; 5 EM Normandie Business School, Metis Lab, 64 rue du Ranelagh, 75016 Paris, France; 6 Excelia Business School, CEREGE (AE 1722), 102 rue de Coureilles, 17024 La Rochelle Cedex 1, France

**Keywords:** Whistleblowing, Perceived organizational protection, Public service motivation, Perceived seriousness of wrongdoing, Education of whistleblowing

## Abstract

Whistleblowers have significantly shaped the state of contemporary society; in this context, this research sheds light on a persistently neglected research area: what are the key determinants of whistleblowing within government agencies? Taking a unique methodological approach, we combine evidence from two pieces of fieldwork, conducted using both primary and secondary data from the US and Indonesia. In Study 1, we use a large-scale survey conducted by the US Merit Systems Protection Board (MSPB). Additional tests are conducted in Study 1, making comparisons between those who have and those who do not have whistleblowing experience. In Study 2, we replicate the survey conducted by the MSPB, using empirical data collected in Indonesia. We find a mixture of corroboration of previous results and unexpected findings between the two samples (US and Indonesia). The most relevant result is that perceived organizational protection has a significant positive effect on whistleblowing intention in the US sample, but a similar result was not found in the Indonesian sample. We argue that this difference is potentially due to the weakness of whistleblowing protection in Indonesia, which opens avenues for further understanding the role of societal cultures in protecting whistleblowers around the globe.

**Supplementary Information:**

The online version contains supplementary material available at 10.1007/s10551-022-05089-y.

## Introduction

A number of government scandals that have emerged in the news media over the past several years have involved whistleblowers who spoke out against perpetrators of wrongdoing, leading to such wrongdoing being widely recognized by both the public and stakeholders. For instance, evidence that whistleblowers played an important role in letting the world know about the gravity of the coronavirus outbreak in China reveals the unique way in which whistleblowers are shaping contemporary society. Another example is the affair known as the Trump-Ukraine scandal, which occurred in the US and involved an officer of the Central Intelligence Agency (CIA) who reported an abuse of power in the form of an attempt to encourage an investigation into Joe Biden, Trump’s political opponent in the 2020 US presidential election. Another famous instance of whistleblower activity is the case of Edward Snowden, who leaked a number of classified National Security Agency (NSA) documents that were meant to be kept secret (Archambeault & Webber, 2015; Latan et al., 2018).

However, a recent study conducted by the Association of Certified Fraud Examiners (ACFE) in 2020 reported that occupational fraud occurring in government and the public administration sector has increased dramatically, especially cases of corruption, followed by white collar crime, conspiracy, money laundering, abuse of power and others (ACFE, 2020). To date, little attention has been devoted to studying this area. Previous studies by Roberts et al. (2011) have provided a guide for managing internal reporting of wrongdoing in the public sector, while the work of Brown and Lawrence (2017) has reported on the strength of organizational processes for responding to staff wrongdoing concerns in the public sector. However, the persistent gap regarding the determinants of whistleblowing in government agencies remains to be fully explored (Mesmer-Magnus & Viswesvaran, 2005; Miceli & Near, 2005). Given that whistleblowing plays a pivotal role in facilitating the reform of government agencies and is often seen as consistent with serving the public or society at large (Caillier, 2017b; Cho & Song, 2015), it is important to investigate the relevant factors that encourage whistleblowers to speak up upon observing wrongdoing. Therefore, this research aims to fill this persistent gap and examines the determinants of whistleblowing within government agencies.

As highlighted in a number of recent studies synthesizing the relevant literature (Culiberg & Mihelič, 2017; Gao & Brink, 2017), there is a large number of existing studies that have investigated individual, situational and organizational factors associated with whistleblowing in the private sector and for-profit organizations (e.g., attitude, personal responsibility, sense of morality, perceived seriousness of wrongdoing, motivation to obtain monetary reward, organizational support, retaliation, wrongdoer power, etc.). Nevertheless, it remains unclear whether these factors will have similar or different effects in relation to blowing the whistle when applied in the public sector and non-profit organizations (Cassematis & Wortley, 2013; Nayır et al., 2018; Scheetz & Wilson, 2019). Furthermore, as indicated in previous studies, there are several critical missing links regarding the relationships between these variables that have not yet been studied thoroughly, which has resulted in a lack of insight in this area and calls for further investigation. As far as we are aware, little attention has been paid by whistleblowing scholars to the overlapping nature of factors such as perceived organizational protection (POP), public service motivation (PSM), perceived seriousness of wrongdoing (PSW) and whistleblowing education (WHE) in influencing observers to speak up about misconduct within government agencies.

Although whistleblowers are often regarded as ‘heroes’ for defending the public interest, they are not infrequently also considered ‘traitors’ for revealing wrongdoing in an organization. A study conducted by Miceli and Near (2013) reports that the involvement of whistleblowers in uncovering misconduct in government agencies has tended to increase over time (Miceli & Near, 2005). Unfortunately, retaliation against whistleblowers has followed the same pattern (Near & Miceli, 2016), with most observers of wrongdoing admitting that they have experienced retaliation. Several studies have documented retaliation against whistleblowers, with consequences ranging from mild to severe, such as being treated unfairly, bullying from co-workers, experiencing verbal harassment and being laid off from work, all of which disturb the mental health of whistleblowers (Latan et al., 2021; Park & Lewis, 2018; Rehg et al., 2008; van der Velden et al., 2019). However, one factor that has not been well studied with regard to mitigating such retaliation is organizational protection (Chordiya et al., 2020). We define perceived organizational protection (POP) as the efforts made by an organization to protect its members from various potential threats when they have decided to blow the whistle. On one hand, an observer will feel comfortable and confident in blowing the whistle when he or she believes that they will be protected after speaking out. On the other hand, when protection is weak or non-existent, an observer may choose to remain silent when considering the potential risks that threaten his or her personal and professional life (Izraeli & Jaffe, 1998; Latan et al., 2021; MacGregor & Stuebs, 2014). Hence, POP can be seen as a security system which increases whistleblowing intention (WBI).

Furthermore, there are other related questions which arise, such as why whistleblowers decide to sacrifice themselves for the public interest, and what motivates them to expose wrongdoing in government agencies? According to Roberts (2014), motivational factors related to the public interest are prominent within government agencies; this includes public service motivation (PSM) and desire to help victims as a result of the perceived seriousness of wrongdoing (PSW) (e.g., fraud, theft, breaches of code of conduct, misuse of allowances or falsification of records). We define PSM as an individual’s orientation toward providing services to people with the aim of serving the public and the wider community. With regard to whistleblowing, PSM can trigger an individual to reveal wrongdoing when it is related to others’ survival. PSM is often associated with an altruistic motive that plays a pivotal role in explaining the intention behind whistleblowing (Caillier, 2017b; Cho & Song, 2015; Ugaddan & Park, 2019). In certain situations, PSM encourages observers to sacrifice themselves for the public good. We define PSW as an observer’s assessment of the magnitude of the consequences generated by illegal, immoral or illegitimate practices (Latan et al., 2021; Rehg et al., 2008). In this regard, the higher the potential impact of wrongdoing on the wider community, the higher the likelihood of observers speaking up. More precisely, whistleblowers often speak up about misconduct which is deemed to have a significant negative impact on the public (e.g., to preserve valuable resources, protect people’s rights and lives or enforce the rule of law). In other words, more serious wrongdoing has greater potential to be reported.

According to the ACFE (2020) report, whistleblowing education (WHE) has received little attention from stakeholders in various organizations, including government agencies. This suggests the possibility that an observer who has witnessed misconduct in the workplace may not know how or to whom to report it (Culiberg & Mihelič, 2017; Vandekerckhove & Lewis, 2012). As Caillier (2017a) argues, scant attention has been devoted to dealing with WHE, and it is still unclear how this factor relates to the intention to blow the whistle. We argue that the lack of WHE has serious implications for whistleblowers’ understanding (WHU) and intention to report wrongdoing. In addition, WHE is considered a vehicle that speeds up the whistleblowing process. WHE can help observers when faced with ethical dilemmas; that is, when wrongdoers hold positions of power in organizational structures (such as supervisors or top-level management). In this context, WHE guides observers regarding how to report their findings without fear of retaliation (e.g., using anonymous channels). Therefore, the existence of WHE has the potential to trigger observers to blow the whistle within government agencies.

Motivated by the aforementioned context, we conducted two original field studies, using employees working for government agencies as a sample. We took samples from two countries—the US and Indonesia—with the aim of potentially increasing the generalizability of our findings. In Study 1, we used data from a large-scale survey conducted by the US Merit Systems Protection Board (MSPB). The aim of Study 1 is to empirically test the determinants of whistleblowing in US government agencies (i.e., POP, PSM, PSW and WHE). Specifically, the MSPB data allow us to examine relationships between variables that have not been explored in previous studies. As has been shown in previous studies in this field (Miceli & Near, 1984, 1989, 2002), the MSPB survey has significant consequences for the reform of government agencies in the US. In Study 2, we replicated Study 1’s format, in order to examine relationships between variables that have not been explored by previous studies using primary data from an Indonesian sample. We found a slight difference between the two sample groups, indicating unexpected findings which can be explained by differences in societal culture and whistleblowing protection acts (WPA).

Our study extends the state-of-the-art research in the field of whistleblowing and provides new insight for this body of knowledge in two ways. First, our research broadens the scope of whistleblowing in the fields of government and public administration, as reported by several previous scholars (Brown & Lawrence, 2017; Miceli & Near, 2002; Roberts et al., 2011). Specifically, this is one of the first empirical studies to consider the latest MPBS survey in examining the relationships between variables using a very large sample size. To our knowledge, recent studies that have used datasets from the MPBS survey are relatively scarce. We note that studies by Ugaddan and Park (2019), Dungan et al. (2019), Caillier (2017ab), and Cho and Song (2015) have used the MBPS survey; however, these works do not fully explore its potential for exploring relationships between variables. For example, Dungan et al. (2019) and Caillier (2017a) only consider ‘simple relationships’ between variables (i.e., between predictors and outcomes). In addition, the studies by Ugaddan and Park (2019), Caillier (2017b) and Cho and Song (2015) only consider a selection of variables individually.

Second, the present research does not depend on a single study. Based on our best knowledge, the use of multiple studies in whistleblowing research is relatively rare. In contrast to previous studies, which rely solely on the MPBS survey, our study uses two field studies to enrich our findings (Miceli & Near, 2002). Therefore, our results provide external validity and a higher potential for the generalization of findings. In addition, this work also answers recent calls by Vandekerckhove et al. (2014) and Latan et al. (2021) to conduct cross-cultural comparative studies; in our case between the US and Indonesia.

The remainder of this paper is organized as follows. The next section presents the theoretical background and development of hypotheses, followed by the research methodology. Following this, the empirical results are presented. Finally, the results are discussed and implications for both academics and practitioners are given.

### Theoretical Background and Development of Hypotheses

#### Whistleblowing in the Public Sector

Recently, whistleblowing has come to receive attention from many organizations, including governments and the public administration sector. Although previous studies have noted the virtuousness of whistleblowing and its positive impacts for society at large (Apaza & Chang, 2017; Lewis et al., 2014; Miceli et al., 2008; Vaughn, 2012), recent developments indicate that ‘blowing the whistle’ has not been an easy feat in the public sector (Brink et al., 2017; Miceli & Near, 2013). Taking whistleblowing action against government agencies, who are some of the largest employers and also those who hold the most power in a given country, is undoubtedly not always an easy decision to make (Lewis, 2015; Vandekerckhove & Lewis, 2012). However, the important role played by whistleblowing in the public sector has several arguments in its favor. First and foremost, within a good system of governance, the public sector can be the most important part of a country’s economy and affects the overall life of the society. Therefore, disclosure of wrongful activities in public sector organizations has the potential benefit of saving people’s lives. In addition, this type of action helps to stop the damage caused by wrongdoing and restore public trust. In some cases, whistleblowing actions can help Presidents, Congresses, agency leaders and/or other decision makers to improve existing systems and identify weaknesses in government agencies. Second, since the public sector is involved in providing services to citizens, disclosure of illegal, immoral and illegitimate acts helps to improve the effectiveness of services (Miceli & Near, 2013). In this regard, such disclosure can mean protecting millions of dollars and reducing service costs to create good governance.

The public sector has certain specific characteristics that should be taken into account when understanding the act of whistleblowing. This sector tends to be more centralized and to have a more hierarchical management structure than the private sector. This characteristic may influence the willingness of public sector employees to speak out against wrongdoing. Another feature of the public sector is the degree of political control over this sector, which may influence bureaucratic behaviors and thus, perhaps, public employees’ whistleblowing decisions. Thus, it is important to conduct research considering the public sector, due to its particular characteristics (Lee, 2020).

As Miceli and Near (2013) argue, the whistleblowing process in the public sector may involve a different route and scope from the private sector. However, based on a broad definition, whistleblowing constitutes the disclosure by members of an organization (including former members and job applicants) of illegal, immoral, or illegitimate practices (including omissions) by the employer, to persons or organizations who may be able to effect action (Near & Miceli, 1985). We argue that the above definition can be applied in both the private and public sectors. In the public sector, the whistleblowing process may involve many stages, and the disclosure of wrongdoing in this sector is specifically regulated by federal law and controlled by the relevant authorities. Furthermore, the scope of wrongdoing may differ in the public sector, given the differences in workplace activity. For example, according to the ACFE (2020) report, misconduct that occurred in the private sector included the misappropriation of assets and fraudulent financial statements. Meanwhile, misconduct that occurred in the public sector included corruption, embezzlement, abuse of power and others. In certain instances, governmental dishonesty or the illegal behavior of government agencies may in fact encourage whistleblowing action.

On the other hand, whistleblowing in the context of the public sector is often associated with a prosocial perspective; that is, behavior which is intended to benefit others as well as oneself (Alford, 2001; Dozier & Miceli, 1985).

Different countries have taken different approaches toward incentivizing the act of speaking out. For example, there are more than 40 pieces of legislation concerning whistleblowing provisions across many jurisdictions in the US, showing that whistleblowing is a widespread cultural phenomenon in this country. However, in other countries, such as Indonesia, there is a lack of law enforcement related to retaliation against whistleblowers. In addition, in countries such as Indonesia, whistleblower protection systems and laws relating to whistleblowing have not been fully regulated. Although government agencies in Indonesia do already have a whistleblowing system in place, it is limited to internal cases. Thus, it is pertinent to consider whether the whistleblower protection measures in place in a given country may relate to whistleblowing intention (WBI). In other words, it is relevant to assess the situation in different countries in terms of whistleblower protection and cultural aspects of the sample context, as in this article.

#### Perceived Organizational Protection Affecting Whistleblowing

According to the prosocial organizational behavior model (Dozier & Miceli, 1985) and the social information processing model (Gundlach et al., 2003), whistleblowers will go through several considerations before deciding whether or not to blow the whistle (Latan et al., 2019, 2021; Near & Miceli, 2011). Often, the whistleblower’s attention is directed toward the potential benefits and threats arising from the act of blowing the whistle. Recently, more attention has been paid to threats and reprisals against whistleblowers. As noted by Rehg et al. (2008), retaliation against whistleblowers is common, and is a form of revenge by an organization or wrongdoer. Given this situation, observers often feel anxious, insecure or depressed as a result of disclosing wrongdoing within an organization. To mitigate this impact, scholars have recently called for increased perceived organizational protection of whistleblowers and research into how these risks or threats can be minimized. A study conducted by Chordiya et al. (2020) concludes that perceived organizational protection for whistleblowers is driven by the ethical climate, legal awareness, ethical leadership and structural provisions. When organizations have a code of ethics, training related to ethics and ethical leadership, this leads to the creation of an ethical climate in the workplace that fosters organizational protection for whistleblowers. In addition, ethical awareness related to whistleblowing law also helps in removing these obstacles. Under such conditions, an observer will feel protected in the act of blowing the whistle and feel comfortable and confident in revealing wrongdoing in the organization.

As Alleyne et al. (2018) and Latan et al. (2018) argue, organizational support encourages observers to speak up upon observing wrongdoing. In addition to motivating observers, organizational support also plays an important role in determining how wrongdoing is reported. Under highly protected conditions, observers can report wrongdoing through internal channels. Conversely, in conditions of weak protection, observers usually choose external or anonymous channels (Alleyne et al., 2018; Latan et al., 2018). Hence, the crucial role of POP is needed in preventing harmful behavior by organizations. We argue that the existence of POP will influence whistleblowers’ understanding (WHU) in selecting reporting channels and this will encourage their intention to be involved in whistleblowing. A study conducted by Cho and Song (2015) indicates that POP has a positive effect on WBI. Prior studies from Ugaddan and Park (2019) and Caillier and Sa (2017) have found that ethical leadership and organizational justice have a positive effect on WHU and intention to report wrongdoing. Other studies by Alleyne et al. (2018) and Latan et al. (2018) report that organizational support has a positive effect on the WBI of public accountants. Based on the above discussion, our concomitant hypotheses are:

##### H1a

Perceived organizational protection has a positive effect on whistleblowing understanding.

##### H1b

Perceived organizational protection has a positive effect on whistleblowing intention.

##### H1c

Perceived organizational protection has a positive indirect effect on whistleblowing intention through whistleblowing understanding.

#### Public Service Motivation Affecting Whistleblowing

PSM has been widely defined as an individual’s predisposition to respond positively with regard to the public interest; this motive is commonly found in government agencies (Perry & Wise, 1990). PSM is generally associated with an altruistic motivation to serve the public interest, which is different from a prosocial perspective (Perry et al., 2010). Therefore, employees with high levels of PSM are less dependent on monetary rewards, while showing a higher loyalty toward bureaucracy (Caillier, 2017b; Cho & Song, 2015). Several studies have documented the relationship between PSM and public employees’ attitudes to work, for instance, job satisfaction, organizational citizenship behavior, commitment to the organization and commitment to society at large (Christensen et al., 2017; Liu & Perry, 2016). According to Perry and Wise (1990), PSM can be divided into three categories of motives: rational, norm-based and affective.

First, rational motives involve actions grounded in individual utility maximization (e.g., capability to blow the whistle). The concept of utility is here associated with the understanding and rationalizing individuals undertake before taking action. Second, norm-based motives refer to actions generated by efforts to conform to norms (e.g., revealing wrongdoing to uphold social justice). These motives generally relate to individual beliefs and values held. Finally, affective motives refer to behavioral triggers that are grounded in emotional responses to various social contexts (e.g., moral anger related to social importance). This category relates to individual feelings when faced with challenging ethical situations.

Perry et al. (2010) revisit the above motivational bases of public service and conclude that PSM reflects self-sacrifice, altruism and prosocial behaviors. In relation to the act of whistleblowing, PSM can trigger an individual to react after observing wrongdoing. On the one hand, PSM invokes a sense of personal responsibility, adherence to ethical norms and the desire of individuals to enact virtuous values. On the other hand, whistleblowers often sacrifice themselves to achieve a higher purpose; that is, serving the public interest, regardless of whether or not they will suffer reprisals. Therefore, PSM facilitates WHU and individuals’ behavior in response to wrongdoing. Several previous studies have indicated a positive relationship between PSM and various outcomes (Christensen et al., 2017). Specifically, Cho and Song (2015) found a positive relationship between PSM and intention to report wrongdoing in government agencies. Another study by Caillier (2017b) found a positive relationship between PSM and whistleblowing intentions, mediated by the seriousness of wrongdoing. Based on the above discussion, our concomitant hypotheses are:

##### H2a

Perceived seriousness of wrongdoing has a positive effect on whistleblowing understanding.

##### H2b

Perceived seriousness of wrongdoing has a positive effect on whistleblowing intention.

##### H2c

Perceived seriousness of wrongdoing has a positive indirect effect on whistleblowing intention through whistleblowing understanding.

#### Perceived Seriousness of Wrongdoing Affecting Whistleblowing

Miceli et al. (2008) argue that the whistleblowing process goes through three phases. The first phase begins with an individual observing activities that are considered questionable and labeling them as wrongful. At this stage, the observer will make an assessment of suspected wrongful activities based on moral standards. In the second phase, once the wrongful activity has been established, the observer will react and respond to it. Usually, an observer will consider the seriousness of the wrongdoing and how wide-ranging the consequences and the potential harm to victims and society at large are. In this regard, before taking whistleblowing action in the third phase, the observer will make an assessment of whether an activity or behavior can be classified as wrongful and/or harmful; this aims to measure the degree of seriousness of the wrongdoing (Ayers & Kaplan, 2005; Rehg et al., 2008). Finally, the observer decides to report their findings through the available reporting channels. The selection of reporting channels will be determined by the degree of seriousness of the wrongdoing, and is therefore related to WHU. In fact, wrongful activities that are considered very serious tend to be reported anonymously, in order to avoid retaliation.

For example, Edward Snowden, who leaked confidential documents from the NSA, considered the seriousness of wrongdoing of the illegal practices he had witnessed (i.e., telephone tapping) and the widespread harmful consequences for society before deciding to blow the whistle. In line with this, we argue that greater seriousness of wrongdoing increases the likelihood of whistleblowing. Greater seriousness of wrongdoing creates greater potential harm, and because of this, it is more likely that a decision will be taken to act on the situation (Keil et al., 2018; Latan et al., 2021). To support this logic, several previous studies have indicated that seriousness of wrongdoing tends to encourage potential whistleblowers to speak up (Ayers & Kaplan, 2005; Keil et al., 2018; Latan et al., 2021). Moreover, PSW can be viewed as based on the magnitude of the consequences (Alleyne et al., 2017; Chen & Lai, 2014)—a component of moral intensity related to the ethical decision-making process. The magnitude of the consequences can be understood as the amount of loss or damage that will result from the wrongdoing. In the face of challenging ethical situations, the magnitude of the consequences often becomes a determinant in ethical decision-making (e.g., deciding to blow the whistle). Several previous studies have found a positive relationship between PSW and intention to report wrongdoing (Andon et al., 2018; Caillier, 2017b; Keil et al., 2018; Latan et al., 2021; Near & Miceli, 1986). Notably, a study by Casal and Bogui (2008) found that an observer would prefer to remain within the organization and blow the whistle, rather than leave the organization after perceiving serious wrongdoing. Based on the above discussion, our concomitant hypotheses are:

##### H3a

Perceived seriousness of wrongdoing has a positive effect on whistleblowing understanding.

##### H3b

Perceived seriousness of wrongdoing has a positive effect on whistleblowing intention.

##### H3c

Perceived seriousness of wrongdoing has a positive indirect effect on whistleblowing intention through whistleblowing understanding.

#### Whistleblowing Education Affecting Whistleblowing

According to organizational support theory (Eisenberger et al., 1986), organizations generally care about the welfare of their employees, value their work efforts and want to meet their social-emotional needs with assurance that aid will be available from the organization when it is needed. There are numerous examples of organizational support, including empowerment, top management commitment, various programs related to career advancement, training and workshops, as well as whistleblowing education (WHE). Organizational support aims to make work practices effective and help those in challenging ethical situations. While a plethora of studies has proven the relationship between organizational support and a number of desirable outcomes, such as individual performance or job satisfaction, it has also been identified as an explanatory variable for the intention behind whistleblowing (Alleyne et al., 2018; Latan et al., 2018). More specifically, the manifestation of organizational support in government agencies relates to WHE. In general, government agencies that have a WHE program educate their employees about how to report wrongdoing. This includes learning about the reporting channels available to them, together with the pros and cons of each option chosen. In addition, WHE also informs employees about relevant whistleblower protection acts (WPA) and associated laws.

Furthermore, Cho and Song (2015) argue that WHE can provide a road map to the whistleblowing process when someone decides to blow the whistle. With regard to whistleblowing, WHE can affect understanding of whistleblowing and related decisions, which can in turn trigger an individual to blow the whistle. When perceived organizational support is received by employees through WHE and disclosure of wrongdoing is fully supported, this enhances the likelihood of employees engaging in whistleblowing. Several previous studies have found a positive relationship between WHE and intention to report wrongdoing (Caillier, 2017a; Cho & Song, 2015; Near & Miceli, 1986). In fact, a study by Jeon (2017) indicates that whistleblowing education is effective for both channels—that is, internal and external—and encourages employees to engage with whistleblowing. Based on the above discussion, our concomitant hypotheses are:

##### H4a

Whistleblowing education has a positive effect on whistleblowing understanding.

##### H4b

Whistleblowing education has a positive effect on whistleblowing intention.

##### H4c

Whistleblowing education has a positive indirect effect on whistleblowing intention through whistleblowing understanding.

#### Whistleblowing Understanding Affecting Whistleblowing

We define WHU as the set of knowledge possessed by individuals or observers about everything related to the process of whistleblowing, its reporting and impact, which helps them in deciding whether or not to blow the whistle. An observer who possesses good WHU knows how to respond to wrongdoing (Miceli et al., 2008; Vaughn, 2012). When wrongdoing has the potential to threaten the whistleblower, WHU works to minimize this impact. In such cases, reporting of misconduct can be made anonymous. Moreover, the observer can request protection from the relevant authorities. A person who has a high level of WHU usually does not experience difficulties when faced with wrongdoing. Generally, he or she knows how to behave, which therefore promotes the likelihood of engaging in whistleblowing. Meanwhile, a person with a lower level of WHU may experience confusion when faced with a challenging ethical situation. In this situation, he or she does not know what action to take when observing wrongdoing, and therefore may choose to remain silent (Latan et al., 2021; MacGregor & Stuebs, 2014). A study conducted by Dungan et al. (2019) found that WHU increased employees’ intention to blow the whistle in government agencies. Based on the above discussion, our fifth hypothesis is:

##### H5

Whistleblowing understanding has a positive effect on whistleblowing intention.

**Fig. 1:**
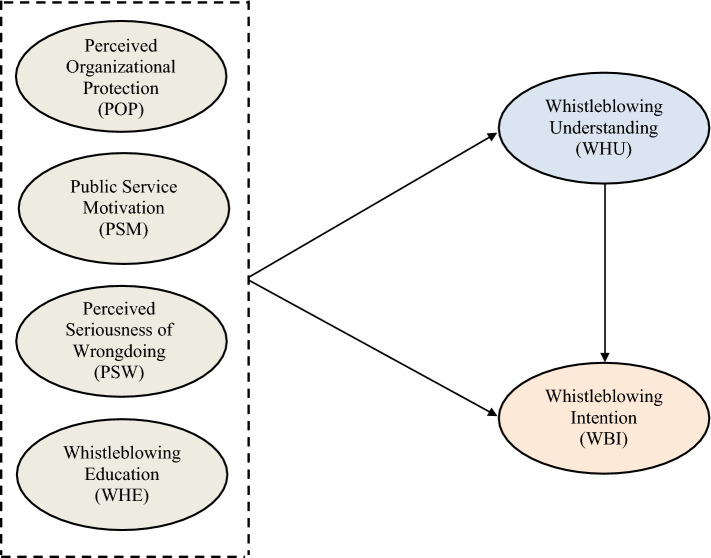
Theoretical framework for understanding whistleblowing intention

Figure 1 portrays the research framework empirically tested in this work.

Whistleblowers have significantly shaped the state of contemporary society; in this context, this research sheds light on a persistently neglected research area: what are the key determinants of whistleblowing within government agencies? Taking a unique methodological approach, we combine evidence from two pieces of fieldwork, conducted using both primary and secondary data from the US and Indonesia. In Study 1, we use a large-scale survey conducted by the US Merit Systems Protection Board (MSPB). Additional tests were conducted in Study 1, making a comparison between those who have and those who do not have whistleblowing experience. In Study 2, we replicate the survey conducted by the MSPB, using empirical data collected in Indonesia. The following sections present each of these two studies.

## Study #1

### Sample and Data Collection

In Study 1, we used the dataset of the 2010 Merit Principles Survey (MPS) conducted by the US Merit Systems Protection Board (MSPB). These data are archival in nature and available in the public domain (https://www.mspb.gov/studies/surveys.htm). For details on the survey and the sampling frame, see Appendix A in the supplementary material, available online.

At the end of the data collection process, MSPB had received 43,162 returned questionnaires; 1142 of these were excluded due to being invalid, giving a final response rate of 58.53%. According to Near and Miceli (2008), the response rate from MPS surveys over time has been very high, and this level meets the rule of thumb for the minimum response rate recommended by various studies (Dillman et al., 2014; Holtom et al., 2022). We conducted preliminary tests to ensure these data are free from biases such as non-response bias and common method variance (CMV). We performed an independent *t*-test, comparing two waves of responses—early and late responders—with the assumption that the late responders represent employees who did not respond to the survey (Fulton, 2018). As shown in Table 1, our results found no significant differences (*p* > 0.05) in either Levene’s test or the equality of means test for the variables tested. We can thus conclude that non-response bias is not a threat to our analysis. In addition, we examined common method variance (CMV), which often occurs when using the self-reporting technique. We used the marker variables approach and compared the goodness-of-fit indices of the model with or without marker variables through confirmatory factor analysis (CFA) (Williams et al., 2010). We found that the marker model showed poor fit and did not correlate with the main constructs in the model. Therefore, we conclude that CMV does not occur in our case. However, we acknowledge that these biases may still exist, even though we did not detect them at this time.

**Table 1:** Assessment of non-response bias

Construct	US		Indonesia	
	Levene’s test	Sig. *t*-test	Levene’s test	Sig. *t*-test
Perceived organizational protection (POP)	0.388	0.870	0.518	0.498
Public service motivation (PSM)	0.856	0.445	0.530	0.375
Perceived seriousness of wrongdoing (PSW)	0.175	0.341	0.959	0.541
Whistleblowing education (WHE)	0.906	0.544	0.753	0.289
Whistleblowing understanding (WHU)	0.655	0.506	0.136	0.960
Whistleblowing intention (WBI)	0.417	0.291	0.703	0.229

We summarize the profile of respondents as shown in Table 2. The largest groups of respondents are those with experience in their role of between 4 and 11 years (25.58%), are in non-supervisory roles (40.91%), and have a bachelor’s degree level of academic education (37.66%).

**Table 2:** Profile of respondents

Demographic variable	US		Indonesia	
	Freq (*f*)	Perc (%)	Freq (*f*)	Perc (%)
Work experience				
Under 4 years	1.329	10.12	13	9.85
4–11 years	3.359	25.58	51	38.64
12–19 years	2.381	18.13	43	32.58
20–27 years	3.335	25.39	15	11.36
28–35 years	1.985	15.11	4	3.03
More than 35 years	744	5.67	6	4.54
Supervisory status				
Non-supervisor	5.373	40.91	96	72.73
Team leader	1.729	13.17	14	10.61
Supervisor	3.578	27.24	13	9.85
Manager	2.317	17.64	6	4.54
Executive	136	1.04	3	2.27
Academic qualifications (level of education)				
Less than high school or high school or equivalent (GED)	872	6.64	4	3.03
Some college credits (no degree) or associate’s college degree	3.686	28.07	8	6.06
Bachelor’s degree	4.946	37.66	64	48.48
Master’s degree	2.538	19.33	32	24.24
Professional degree (e.g., M.D, D.D.S, etc.)	662	5.04	20	15.15
Doctorate degree (Ph.D)	429	3.26	3	2.27
Pay system				
General schedule	10.228	77.88	106	80.3
Wage grade	1.203	9.16	14	10.61
Executive (senior executive service)	94	0.72	3	2.27
Other	1.608	12.24	9	6.82

Freq = frequency, Perc = percentage

### Questionnaire Design and Measures

We identified 23 items for measuring the variables in our proposed model. Since the MSPB survey involves many factors related to the workplace, not only whistleblowing-related aspects (e.g., job satisfaction, loyalty, job barriers, job recognition, leadership, etc.) (Dungan et al., 2019), we excluded these irrelevant items from the analysis. A complete list of items used in this study is depicted in Tables 3 and 4. We note that the 23 items selected were spread across a number of sections in the 2010 MPS questionnaire.

**Table 3:** Measurement model assessment of perceived organizational protection, public service motivation and perceived seriousness of wrongdoing

Indicator/item	Code	PCA	Mean	SD	FL	AVE	*α*	*ρ*_*c*_
*(A) Perceived organizational protection (POP)*						0.913	0.952	0.969
My organization protects employees against reprisals for whistleblowing	POP1	0.952	3.661	0.976	0.953			
My organization protects employees against reprisals for exercising a grievance, complaint, or appeal right	POP2	0.967	3.675	0.991	0.967			
My organization protects employees against arbitrary action	POP3	0.947	3.647	0.964	0.947			
*(B) Public service motivation (PSM)*						0.509	0.759	0.838
Meaningful public service is important to me	PSM1	0.711	4.364	0.667	0.705			
I am not afraid to go to bat for the rights of others, even if it means I will be ridiculed	PSM2	0.665	4.224	0.754	0.674			
I am prepared to make enormous sacrifices for the good of the agency	PSM3	0.757	3.736	0.955	0.757			
I am often reminded by daily events about how dependent we are on one another	PSM4	0.674	3.916	0.844	0.698			
Making a difference in society means more to me than personal achievement	PSM5	0.758	3.867	0.867	0.730			
*(C) Perceived seriousness of wrongdoing (PSW)*						0.684	0.734	0.806
The activity might endanger people’s lives	PSW1	0.856	4.879	0.494	0.645			
The activity was something I considered serious in terms of costs to the Government	PSW2	0.856	4.531	0.712	0.976			

PCA = principal component analysis, FL = factor loading, SD = standard deviation, AVE = average variance extracted, α = Cronbach’s Alpha, ρ_c_ = composite reliability

**Table 4:** Measurement model assessment of whistleblowing education, whistleblowing understanding and whistleblowing intention

Indicator/item	Code	PCA	Mean	SD	FL	AVE	*α*	*ρ*_*c*_
*(A) Whistleblowing education (WHE)*						0.874	0.928	0.954
My agency has educated me about the purpose of the office of the inspector general	WHE1	0.900	3.681	1.040	0.907			
My agency has educated me about how I can anonymously disclose wrongdoing	WHE2	0.954	3.651	1.039	0.951			
My agency has educated me about what my rights would be if I disclosed wrongdoing	WHE3	0.950	3.659	1.028	0.946			
*(B) Whistleblowing understanding (WHU)*						0.704	0.859	0.905
The US office of the special counsel (OSC)	WHU1	0.847	2.329	0.914	0.841			
The government accountability office (GAO)	WHU2	0.897	2.563	0.916	0.892			
My agency’s office of the inspector general (OIG)	WHU3	0.842	2.904	0.912	0.860			
The occupational safety and health administration (OSHA)	WHU4	0.767	2.896	0.873	0.757			
*(C) Whistleblowing intention (WBI)*						0.704	0.916	0.934
My supervisor	WBI1	0.837	3.844	1.110	0.852			
A higher level supervisor	WBI2	0.860	3.884	1.110	0.871			
A coworker (in my work group)	WBI3	0.874	4.039	0.949	0.882			
A Federal employee outside my work group	WBI4	0.86	4.208	0.881	0.851			
A contractor or vendor	WBI5	0.78	4.410	0.821	0.763			
A political appointee in my agency	WBI6	0.824	4.171	1.018	0.811			

PCA = principal component analysis, FL = factor loading, SD = standard deviation, AVE = average variance extracted, α = Cronbach’s Alpha, ρ_c_ = composite reliability

To measure perceived organizational protection (POP), 3 items were selected to reflect this variable. A 5-point Likert scale, ranging from 1 = “strongly disagree” to 5 = “strongly agree,” was used to measure this construct. For instance, respondents were asked “please indicate your level of agreement or disagreement—whether the organization protects employees against reprisal for whistleblowing” and so on. Furthermore, public service motivation (PSM) and perceived seriousness of wrongdoing (PSW) were measured using 5 and 2 items, respectively. Again, A 5-point Likert scale was used to measure both of these constructs. These scales ranged from 1 = “strongly disagree” to 5 = “strongly agree” and 1 = “unimportant” to 5 = “very important,” respectively. In the same vein, respondents were asked, regarding PSM, items such as “please indicate your level of agreement or disagreement – whether making a difference in society means more than personal achievements.” On the other hand, regarding PSW, respondents were presented with items such as “how important, if at all, would each of the following be in encouraging you to report an illegal or wasteful activity, such as, the activity might endanger people’s lives.”

Meanwhile, the remaining variables were also measured using multiple measurement items. Whistleblowing education (WHE) was measured using 3 items and adopted a 5-point Likert scale from 1 = “strongly disagree” to 5 = “strongly agree.” For example, respondents were asked “please indicate your level of agreement or disagreement – whether your agency has educated you about the purpose of the Office of the Inspector General” and so on. Further, whistleblowing understanding (WHU) was measured using 4 items related to the organization of reporting channels. This time, a 4-point Likert scale was applied, ranging from 1 = “not at all” to 4 = “great extent.” Respondents were asked questions such as “to what extent do you understand the role of each of the following organizations when it comes to responding to reports of wrongdoing.” Finally, whistleblowing intention (WBI) was measured using 6 items and a 5-point Likert scale from 1 = “very unlikely” to 5 = “very likely.” Respondents were asked to indicate “how likely would you be to blow the whistle when the wrongdoer was a supervisor” and so on.

### Data Analysis

A covariance-based structural equation modeling (CB-SEM) method was used to analyze our data and confirm hypothesis testing. We chose CB-SEM based on several key considerations. First, CB-SEM is a second-generation analysis technique that allows us to examine the causal relationships between unobserved variables simultaneously based on theoretical grounds. In this regard, CB-SEM provides various goodness-of-fit indices, including ‘absolute,’ ‘incremental’ and ‘parsimonious’ (Byrne, 2016; Kline, 2016). Second, CB-SEM allows us to perform CFA (Collier, 2020). In addition, CB-SEM provides various choices in terms of estimation methods, such as maximum likelihood (ML) or generalized least squares (GLS) for normal data assumptions, and asymptotically distribution-free (ADF) and full information maximum likelihood (FIML) for non-normal data and missing values. Finally, CB-SEM is a robust approach which produces stable estimates.

Regarding the sample size requested in the CB-SEM estimates, we followed the rule of thumb provided by experts in the field, although we recognize that there is no general consensus. We follow the recommended minimum sample size as given by Byrne (2016) and Kline (2016) to perform the ADF estimation method in CB-SEM, which is not less than 5000 cases. However, when a small sample size (e.g., less than 1000 cases) is used in the ADF estimator, the results generally cannot be trusted, as they tend to be very poor and distorted. Because our case meets this requirement, and it is difficult to obtain normal data distribution in the ML estimator using a very large sample size, we chose to use the ADF method. The ADF estimator makes no distributional assumptions for continuous outcomes. In addition, the ADF estimator will provide accurate results from parameter estimates (i.e., no distorted estimated values and standard errors) and avoid the appearance of Heywood cases (i.e., negative variance). Second, we considered the identification of the model in CB-SEM analysis by calculating the number of distinct values in the sample variance–covariance matrix as equal to or higher than the number of parameters to be estimated (Whittaker & Schumacker, 2022). A model is called ‘identified’ if the degree of freedom is equal to or greater than 1.

Finally, we are reporting the results of our analysis following the guidelines for best practice (Boomsma et al., 2014; Zhang et al., 2021). We have divided this reporting phase into three subprocesses. First, we will report the results of the measurement model through CFA analysis (i.e., factor loadings, average variance extracted, composite reliability, etc.). Second, we will report the results of the structural model through the ADF estimator (i.e., *R*^2^, *f*^2^, goodness-of-fit, critical ratio, etc.). Finally, we will report the results of the robustness checks conducted to strengthen our main findings.

## Results

The AMOS 28.0 program was used to test our research model. According to Byrne (2016), AMOS is preferred because of its simplicity, and generally involves a graphical interface. As mentioned earlier, we used a number of specific settings, such as selecting the ADF estimator and activating the desired output options. We assessed multivariate normality through the critical ratio (CR) values of skewness and kurtosis to justify the use of the ADF estimator. Based on the results of the assessment of normality, we obtained a CR value for kurtosis of 10.647 > 10 and for skewness of − 33.152 < 3. According to Kline (2016), we can thus conclude that our data are not normally distributed. Since our dataset is very large, the multivariate normality assumption is ignored in this regard, thereby justifying the use of the ADF estimator.

Tables 3 and 4 present descriptive statistics (means and standard deviations) of each indicator variable, while Table 5 presents the correlation between variables. Following the guidelines of Bedeian (2014), we confirm that the mean and standard deviation values of the variables in the model do not exceed the maximum and the correlation sign is not reversed. We found no correlation greater than 0.70 for all pairs of relationships between variables, which gives an initial indication that our data are free from collinearity. To support this conclusion, we calculated the variance inflation factor (VIF) for each predictor and found VIF values < 3.3, which meets the rule of thumb threshold (see Table 6).

**Table 5:** Assessment of discriminant validity using Fornell–Larcker criterion, Hetero-Trait Mono-Trait ratio, and correlations

Construct	1	2	3	4	5	6
POP	** − 0.955**	0.113**	0.229**	0.510**	0.204**	0.316**
PSW	0.133 [0.154;111]	** − 0.827**	0.181**	0.095**	0.202**	0.101**
PSM	0.266 [0.285;247]	0.241 [0.267;218]	** − 0.713**	0.243**	0.215**	0.235**
WHE	0.543 [0.557;529]	0.108 [0.129;091]	0.287 [0.305;270]	** − 0.935**	0.234**	0.525**
WBI	0.217 [0.233;200]	0.248 [0.272;224]	0.258 [0.275;240]	0.250 [0.266;234]	** − 0.839**	0.201**
WHU	0.349 [0.364;333]	0.121 [0.143;106]	0.290 [0.308;273]	0.587 [0.600;574]	0.223 [0.239;206]	** − 0.839**

Brackets show the upper and lower bounds of the 95% BCa confidence intervals. Diagonal and bold elements are the square roots of the AVE (average variance extracted). Below the diagonal are the HTMT values. Above the diagonal are the correlations between the constructs

**Correlation is significant at the 0.01 level (2-tailed)

**Table 6:** Structural model assessment

Construct	*R*^2^	*f*^2^	VIF	GoF	Cut-off
Perceived organizational protection (POP)	–	0.015	1.382	CFI = 0.819	Marginal
Public service motivation (PSM)	–	0.032	1.132	IFI = 0.819	Marginal
Perceived seriousness of wrongdoing (PSW)	–	0.115	1.056	NFI = 0.811	Marginal
				GFI = 0.901	Fit
Whistleblowing education (WHE)	–	0.234	1.710	AGFI = 0.873	Fit
Whistleblowing understanding (WHU)	0.367	0.104	1.422	PCFI = 0.696	Fit
Whistleblowing intention (WBI)	0.131	–	–	PNFI = 0.689	Fit
				RMSEA = 0.038	Fit
				RMR = 0.062	Fit

### Measurement Model Assessment

Since the items for each variable were taken separately from the 2010 MPS questionnaire, we conducted a series of factor analyses through principal component analysis (PCA) to test the reliability of these items. Using IBM SPSS 28.0, we obtained a Kaiser–Meyer–Olkin Measure of Sampling Adequacy (KMO-MSA) > 0.50 for each variable in the model, with 1 component extracted. In Tables 3 and 4, the value of factor loadings obtained is greater than 0.50, supporting these items forming a single factor. Thereafter, we conducted a CFA analysis to assess the measurement model, consisting of factor loadings, average variance extracted (AVE), Cronbach’s Alpha (α) and composite reliability (*ρ*_*c*_). We obtained factor loading values for each indicator > 0.708 and AVE > 0.50, respectively, in line with the recommended threshold for convergent validity (Collier, 2020; Garson, 2015). Although a few indicators yielded values slightly below this cut-off, it is still acceptable for strengthening content validity. In addition, we obtained α and *ρ*_*c*_ values both greater than 0.70, which meets the requirements of internal consistency reliability (Garson, 2015). Finally, the GoF index for the CFA model was assessed. We obtained the following values: comparative fit index (CFI) = 0.941 > 0.90, incremental index of fit (IFI) = 0.942 > 0.90, goodness-of-fit index (GFI) = 0.969 > 0.90, adjusted goodness-of-fit index (AGFI) = 0.952 > 0.85, normed fit index (NFI) = 0.937 > 0.90, root mean square error of approximation (RMSEA) = 0.030 < 0.08, and root mean square residual (RMR) = 0.057 < 0.08, which shows the appropriate fit of our CFA model.

Furthermore, we assessed discriminant validity using two approaches. First, we compared the square root of AVE with the correlation between latent variables. This approach is often called the Fornell–Larcker criterion. In Table 5, the diagonal line shows the square root of AVE greater than the correlation, which means that discriminant validity is fulfilled. Second, we assessed the Hetero-Trait-Mono-Trait (HTMT) ratio. HTMT values greater than 0.90 show similarity of measurements between variables, while HTMT values less than 0.85 show the opposite. In Table 5, the HTMT values generated below the diagonal line are less than 0.85, indicating that discriminant validity is met for our measurements.

### Structural Model Assessment

We decided to report several core metrics for evaluating structural models and related parameters. In this regard, we report here the coefficient of determination (*R*^2^) for each endogenous variable, the effect size (*f*^2^) for each predictor in the model and the overall GoF indices, including ‘absolute,’ ‘incremental’ and ‘parsimonious,’ to assess compatibility between our model and data. As shown in Table 6, we obtained *R*^2^ values for the two endogenous variables (in our case WHU and WBI), which were 0.131 and 0.367, respectively. These *R*^2^ values fall into the moderate and large categories, respectively (Cohen, 1988). In addition, Table 6 shows that the *f*^2^ values that we obtained, which highlight the strength of the relationship obtained between an independent variable and a dependent variable, ranged from 0.015 to 0.234 (Rosnow & Rosenthal, 2009). Finally, we assessed the GoF index from the structural model. We obtained the following values: CFI = 0.819, IFI = 0.819, GFI = 0.901, AGFI = 0.873, NFI = 0.811, PCFI = 0.696, PNFI = 0.689, RMSEA = 0.038 and RMR = 0.062. We conclude that our structural model does fit with the data (Kline, 2016; Whittaker & Schumacker, 2022).

### Testing of Hypotheses—Direct Effect

We tested our hypotheses simultaneously through the ADF estimator. To test whether our hypotheses were supported or not, we looked at the sign of the path coefficient (*β*) and the critical ratio (CR) values on the relationships between variables. First, we examined direct effects and found that all hypotheses are supported empirically, as depicted in Table 7. Specifically, we found empirical support for the path relationships POP→WHU and POP→WBI, with beta (*β*) values of 0.038 and 0.080, respectively, and significance at CR = 4.165 > 1.96 and CR = 5.615 > 1.96. In addition, the path relationships PSM→WHU and PSM→WBI were also supported by our findings, with beta (*β*) values of 0.141 and 0.302, respectively, and significance at CR = 11.226 > 1.96 and CR = 14.484 > 1.96. Therefore, we can conclude that H1a, H1b, H2a, and H2b are confirmed. Subsequently, we found that the path relationships PSW→WHU and PSW→WBI, as well as WHE→WHU and WHE→WBI, were fully supported, with beta (*β*) values of 0.016, 0.109, 0.413, and 0.116, respectively, and significance at CR > 1.96. Hence, we can draw the conclusion that H3a, H3b, H4a, and H4b are confirmed. Finally, for the path relationship WHU→WBI, we found the value of *β* = 0.078 and CR = 4.650 > 1.96. Thus, H5 was confirmed in our findings.

**Table 7:** Testing of hypotheses (direct effect)

Structural path	Coef (*β*)	SD	*p* value	CR	Conclusion
POP $\to$ WHU	0.038	0.009	0.000**	4.165**	H1a supported
POP $\to$ WBI	0.080	0.014	0.000**	5.615**	H1b supported
PSM $\to$ WHU	0.141	0.013	0.000**	11.226**	H2a supported
PSM $\to$ WBI	0.302	0.021	0.000**	14.484**	H2b supported
PSW $\to$ WHU	0.016	0.005	0.001**	3.286**	H3a supported
PSW $\to$ WBI	0.109	0.020	0.000**	5.434**	H3b supported
WHE $\to$ WHU	0.413	0.009	0.000**	46.292**	H4a supported
WHE $\to$ WBI	0.116	0.015	0.000**	7.946**	H4b supported
WHU $\to$ WBI	0.078	0.017	0.000**	4.650**	H5 supported

**, *Statistically significant at the 1 percent and 5 percent levels, respectively

Coef (β) = beta coefficient, SD = standard deviation, CR = critical ratio

### Testing of Hypotheses—Indirect Effect

In addition to examining direct effects, we also tested indirect effects to justify the role of a mediating variable (in our case WHU). Following Hayes’s (2022) recommendation for the simple mediation model using the PROCESS procedure, we found the results of indirect effects as depicted in Table 8. We found that the indirect effects pathway was fully supported in our case. Notably, for the POP→WHU→WBI and PSM→WHU→WBI paths, both were significant at CR > 1.96. According to Hayes (2022), the assumption of indirect effects is therefore fulfilled. Additionally, for the PSW→WHU→WBI and WHE→WHU→WBI paths, both were also significant. Consequently, our findings empirically confirmed H1c, H2c, H3c, and H4c.

**Table 8:** Testing of hypotheses (indirect effect)

Structural path	Coef (*β*)	SD	*p* value	CR	Conclusion
POP $\to$ WHU $\to$ WBI	0.030	0.001	0.000**	4.072**	H1c supported
PSM $\to$ WHU $\to$ WBI	0.030	0.001	0.000**	3.955**	H2c supported
PSW $\to$ WHU $\to$ WBI	0.070	0.001	0.000**	5.952**	H3c supported
WHE $\to$ WHU $\to$ WBI	0.340	0.005	0.000**	6.815**	H4c supported

**, *Statistically significant at the 1 percent and 5 percent levels, respectively

Coef (β) = beta coefficient, SD = standard deviation, CR = critical ratio

### Robustness Checks

We conducted two robustness checks to reinforce our main findings. First, we assessed endogeneity bias through the Durban and Wu-Hausman tests with the help of the STATA 17.0 program. We found no endogeneity biases occurring in our case (*p* > 0.05), including inverse causality, sample-selection bias,, and omitted variables (Ullah et al., 2021). These results confirm that our main findings are free from endogeneity bias. Second, we examined the potential for nonlinear relationships between variables. Because CB-SEM generally assumes a linear combination between variables (Garson, 2015), the nonlinear pattern (e.g., quadratic effect) is considered absent. To test for the existence of this nonlinear pattern, we used Ramsey’s regression specification error test (RESET) (Wooldridge, 2020). Based on the results of Ramsey’s test, we found values of *p* > 0.05 for all possible relationships. Hence, we can conclude that our model has been correctly specified (Whittaker & Schumacker, 2022; Wooldridge, 2020).

### Additional Testing

This test aims to identify the difference between those who have and those who do not have whistleblowing experience. Specifically, this test adds to the validity of the findings from Study 1. We classified respondents based on their responses to a number of further question items and compared the differences between them. The respondents were asked “regardless of whether or not it is part of your job, during the last 12 months, did you personally observe or obtain direct evidence of one or more illegal or wasteful activities involving your agency? (*Note*: do not answer “yes” if you only heard about the activity in the media or heard about it as a rumor.)” Based on this question, respondents were divided into three groups. First, respondents who answered “no” were classified as non-observers (*n* = 34.463); that is, those who had not observed wrongdoing. Second, respondents who answered “yes” were asked to “select the one activity that represents the most serious problem you know about” from a list of 10 response categories: stealing federal funds; stealing federal property; accepting bribes or kickbacks; waste caused by ineligible people receiving funds, goods, or services; waste caused by unnecessary or deficient goods or services; use of an official position for personal benefit; waste caused by a badly managed program; unfair advantage in the selection of a contractor, consultant, or vendor; tolerating a situation or practice which poses a substantial and specific danger to public health or safety; and other serious violation of law or regulation.

Third, observers were asked “did you report the activity to any individual or a group?”. Observers who answered “no” were classified as those who did not have whistleblowing experience (*n* = 1.312); that is, those who had observed wrongdoing but did not report it. Finally, observers who answered “yes” were classified as those who have whistleblowing experience (*n* = 1.764); that is, those who had both observed and reported wrongdoing. Furthermore, observers who merely discussed the matter with family members, friends or an unknown party (as indicated by selecting “other”) or mentioned it informally to co-workers did not count as whistleblowers; thereby, these cases were excluded from our analysis (*n* = 601). In addition, there were about 93 remaining respondents who reported that they had observed an illegal activity but did not answer the question about who they reported the activity to; these cases were therefore also excluded from our analysis.

We conducted a multigroup analysis (MGA) to compare our subsamples using PLS path modeling. In this regard, we compared those who have and those who do not have whistleblowing experience. We assessed the measurement invariance of composite models (MICOM) before conducting a PLS-MGA analysis. As depicted in Table 9, the MICOM results indicate that both configural and compositional invariance were established (*p* > 0.05) within step 2 and step 3 of the permutation tests. According to Hair et al. (2022), the measurement invariance of our subsamples is therefore confirmed. Furthermore, PLS-MGA analysis was carried out using non-parametric approaches: PLS-MGA and permutation tests. The PLS-MGA results for the relationships between variables are presented in Table 9. We found that the path coefficients between the two sample groups (no experience vs. experience in whistleblowing) did not differ significantly. Specifically, the results of the PLS-MGA and permutation tests showed no significant difference (*p* > 0.05) between the two samples. In addition, the results of each group did not show significant differences in the findings, with both groups giving similar results. Therefore, we conclude that there is no difference in intention between those who have and those who do not have whistleblowing experience, which strengthens the findings of Study 1.

**Table 9:** PLS-MGA results

Structural path	No experience (*β*)	Experience (*β*)	Diff	95% BCa CI permutation	MICOM	PLS-MGA	Equal Var.	Conclusion
POP $\to$ WHU	0.055^n.s^	0.023^n.s^	0.032	0.136^n.s^	0.733^n.s^	0.138^n.s^	Yes	No difference
POP $\to$ WBI	0.012^n.s^	0.013^n.s^	0.001	0.377^n.s^	0.496^n.s^	0.326^n.s^	Yes	No difference
PSM $\to$ WHU	0.090*	0.142**	0.052	0.175^n.s^	0.250^n.s^	0.187^n.s^	Yes	No difference
PSM $\to$ WBI	0.192**	0.086*	0.106	0.105^n.s^	0.218^n.s^	0.092^n.s^	Yes	No difference
PSW $\to$ WHU	0.034^n.s^	0.044^n.s^	0.010	0.459^n.s^	0.113^n.s^	0.429^n.s^	Yes	No difference
PSW $\to$ WBI	0.204*	0.262**	0.058	0.190^n.s^	0.448^n.s^	0.253^n.s^	Yes	No difference
WHE $\to$ WHU	0.535**	0.459**	0.076	0.115^n.s^	0.473^n.s^	0.100^n.s^	Yes	No difference
WHE $\to$ WBI	0.086*	0.072*	0.014	0.434^n.s^	0.389^n.s^	0.420^n.s^	Yes	No difference
WHU $\to$ WBI	0.022^n.s^	0.029^n.s^	0.007	0.118^n.s^	0.337^n.s^	0.127^n.s^	Yes	No difference

n.s. = not significant, Var = variances, Diff = difference, BCa CI = bias-corrected and accelerated confidence intervals

*p < 0.05 (one-tailed test)

**p < 0.01 (one-tailed test)

## Study #2

### Sample and Data Collection

In order to enhance the generalizability of the findings from Study 1, we collected empirical data in Indonesia, using questionnaire items provided by the 2010 MPS survey. For details on the survey and the sampling frame, see Appendix B in the supplementary material available online.

Ultimately, we received 167 responses by the deadline for returning the questionnaire. From this initial rate of return, 35 were excluded due to being incomplete, giving a final response rate of 24.81%. We ran a t-test to detect non-response bias. As shown in Table 1, we found no significant mean differences (*p* > 0.05) in either Levene’s test or the equality of means test. From these results, we can conclude that non-response bias is not a threat to the validity of our findings. Subsequently, we performed a CFA test with the marker variables technique to detect common method variance (CMV). We found that the marker model did not fit the data and did not correlate with the main constructs. From these results, we can conclude that CMV did not occur in our case. Again, we acknowledge that these biases may still exist, and our data may not be completely free from these biases. A summary of the profile of respondents for Study 2 is depicted in Table 2.

### Measurement Items and Scales

We obtained factor loading values for the POP and PSM constructs greater than 0.708 and AVE values > 0.05, respectively. In addition, the Cronbach’s Alpha (α) and composite reliability (*ρ*_*c*_) values obtained for both are greater than 0.70, which satisfies the validity and reliability of constructs. Meanwhile, for the PSW and WHE constructs, similar results were obtained. The lowest factor loading value for the two constructs is 0.885 > 0.708, with AVE values ranging from 0.789 to 0.817. For α and *ρ*_*c*_, these values range from 0.733 to 0.888. Finally, for the WHU and WBI constructs, we obtained factor loading values for both ranging from 0.736 to 0.932, with the lowest AVE value 0.658 > 0.50. Additionally, for α and *ρ*_*c*_, both values meet the rule of thumb, ranging from 0.824 to 0.941.

### Data Analysis

We employed PLS path modeling for our data analysis. The PLS approach is useful when the research involves complex models with medium sample sizes. This approach is often referred to as an alternative method to structural equation modeling (SEM). In addition, PLS offers several advantages, such as not applying parametric assumptions (often called distribution free) and supporting advanced features. Based on this consideration, we chose PLS as these reasons made it the superior choice in our case. We then calculated the minimum sample requirements for our model and found our sample size to be greater than the recommended minimum of 146 cases (where the minimum absolute significant path coefficient = 1.97, significant level = 0.05 and required power level = 0.80). The results of the PLS analysis will be reported as follows. First, we will report the results of the structural model assessment including path coefficient, *R*^2^ values, *f*^2^, etc. Second, we will report the results of our hypothesis testing, which was conducted using the bootstrapping approach at a 95% confidence interval (CI). Finally, we will provide a robustness test for unobserved heterogeneity to ascertain the main results.

We used the SmartPLS 3 software to analyze our data (Ringle et al., 2015). We implemented a number of specific settings before running this software. In the PLS algorithm settings, we selected the path weighting scheme with the maximum number of iterations set at 300 and a stop criterion of 10^−7^ (= 1.0E−07). In terms of bootstrapping, we used 5,000 subsamples to obtain stability of model estimates through confidence interval methods, namely a bias-corrected and accelerated (BCa) bootstrap. In addition, we set the level of significance to reject the null hypothesis at 5% (one-tailed). The results obtained are described below.

## Results

Since the PLS model estimates the relationship between latent variables by means of linear aggregates of the indicators, the assumption of collinearity among predictors needs to be considered. We obtained VIF values for predictors in the model of less than 3.3 (see Table 10), which indicate that this issue does not occur in our case.

**Table 10:** Structural model assessment

Construct	*R*^2^	Adj. *R*^2^	*f* ^2^	*Q*^2^	VIF	*SRMR*
Perceived organizational protection (POP)	–	–	0.033	–	1.423	–
Public service motivation (PSM)	–	–	0.094	–	2.011	–
Perceived seriousness of wrongdoing (PSW)	–	–	0.072	–	1.420	–
Whistleblowing education (WHE)	–	–	–	–	1.540	–
Whistleblowing understanding (WHU)	0.450	0.433	0.049	0.280	1.819	0.065
Whistleblowing intention (WBI)	0.546	0.528	0.044	0.400	–	0.065

### Structural Model Assessment

An assessment of the structural model for Study 2 is exhibited in Table 10. We obtained *R*^2^ values for the WHU and WBI constructs ranging from 0.450 to 0.546, respectively. According to Cohen (1988), these values fall into the large category. Regarding the magnitude of the variance contributed, we obtained *f*^2^ values ranging from 0.033 to 0.094, as depicted in Table 10. Based on Cohen (1988), these *f*^2^ values are included in the small to moderate category. Finally, we obtained predictive relevance (*Q*^2^) values through the blindfolding procedure for each dependent variable in the model, which were greater than 0. The *Q*^2^ results indicate the PLS model does ‘fit.’ This is supported by the standardized root mean square residual (SRMR) value of 0.065 < 0.08.

### Testing of Hypotheses—Direct Effect

Overall, we found support for our hypotheses for each direct effect in the model as shown in Table 11, apart from the connecting path POP→WBI, which is not supported. For the path relationships POP→WHU, PSM→WHU and PSM→WBI, we found a positive and significant effect with beta (*β*) values of 0.158, 0.308, and 0.267, respectively, and significant at *p* < 0.05 at 95% CI. Therefore, H1a, H2a, and H2b are confirmed. In addition, for the path relationships PSW→WHU, PSW→WBI, WHE→WHU and WHE→WBI, we found a positive and significant effect with beta (*β*) values of 0.228, 0.214, 0.191, and 0.185, respectively, and significance at *p* < 0.05 at 95% CI. Hence, H3a, H3b, H4a and H4b are also confirmed. Finally, regarding the WHU→WBI link, our results reflect a positive and significant relationship, with *β* = 0.191, *p* = 0.019 < 0.05 at 95% CI. Accordingly, H5 is confirmed.

**Table 11:** Testing of hypotheses (direct effect)

Structural path	Coef (*β*)	SD	*p* value	95% BCa CI	Conclusion
POP $\to$ WHU	0.158	0.071	0.013*	(0.037, 0.268)**	H1a supported
POP $\to$ WBI	0.121	0.092	0.094^n.s^	(− 0.023, 0.276)^n.s^	H1b not supported
PSM $\to$ WHU	0.308	0.106	0.002**	(0.139, 0.489)**	H2a supported
PSM $\to$ WBI	0.267	0.102	0.004**	(0.107, 0.440)**	H2b supported
PSW $\to$ WHU	0.228	0.095	0.008**	(0.067, 0.383)**	H3a supported
PSW $\to$ WBI	0.214	0.095	0.012*	(0.053, 0.366)**	H3b supported
WHE $\to$ WHU	0.191	0.089	0.016*	(0.049, 0.345)**	H4a supported
WHE $\to$ WBI	0.185	0.111	0.048*	(0.006, 0.365)**	H4b supported
WHU $\to$ WBI	0.191	0.092	0.019*	(0.041, 0.340)**	H5 supported

**, *Statistically significant at the 1 percent and 5 percent levels, respectively

Coef (*β*) = beta coefficient, SD = standard deviation, BCa CI = bias-corrected and accelerated confidence intervals

^n.s^= not significant

### Testing of Hypotheses—Indirect Effect

We also looked at specific indirect effects on the SmartPLS output in order to assess the role of a mediating variable. Following the guidelines of Hair et al. (2022) for testing the indirect effect in the PLS framework, we obtained the results as shown in Table 12. We found both the path relationships PSM→WHU→WBI and PSW→WHU→WBI to be significant at *p* < 0.05 at 95% CI. Therefore, we can conclude that H2c and H3c are supported. Meanwhile, regarding the indirect effect WHE→WHU→WBI, we found a positive and significant effect, with beta (*β*) values of 0.036 and significant at *p* < 0.05 at 95% CI. Hence, H4c is also supported.

**Table 12:** Testing of hypotheses (indirect effect)

Structural path	Coef (*β*)	SD	*p* value	95% BCa CI	Conclusion
POP $\to$ WHU $\to$ WBI	0.030	0.021	0.077^n.s^	(0.004, 0.048)^n.s^	H1c not supported
PSM $\to$ WHU $\to$ WBI	0.044	0.025	0.042*	(0.014, 0.105)*	H2c supported
PSW $\to$ WHU $\to$ WBI	0.059	0.012	0.018*	(0.009, 0.150)*	H3c supported
WHE $\to$ WHU $\to$ WBI	0.036	0.022	0.046*	(0.012, 0.091)*	H4c supported

**, *Statistically significant at the 1 percent and 5 percent levels, respectively

Coef (*β*) = beta coefficient, SD = standard deviation, BCa CI = bias-corrected and accelerated confidence intervals

^n.s^= not significant

### Robustness Tests

We utilized the finite mixture PLS (FIMIX-PLS) algorithm to test unobserved heterogeneity in order to strengthen our findings. In this process, we assessed the goodness-of-fit (GoF) index for model comparisons. We obtained consistent Akaike’s information criterion (CAIC) values of *k* = 1, as opposed to *k* = 2 or *k* = 3, which indicates that this bias was absent. To confirm this conclusion, we examined modified AIC with factor 4 (AIC_4_), the Bayesian information criteria (BIC) and minimum description length with factor 5 (MDL_5_), which generally works better to determine the number of segments. Our results indicate that there is no difference between these results, which confirms our previous conclusion.

## Discussion and Implications for Theory and Practice

Recently, the government and public administration sectors have faced tremendous challenges to create good governance based on the principles of democracy, transparency and public accountability. Scandals such as abuses of power or severe corrupt practices in government agencies (ACFE, 2020) have affected public trust in general and demoralized civil servants (Miceli et al., 2012). One way to combat these unethical practices in government agencies is through whistleblowing acts. Whistleblowing acts have been proven to be helpful in rebuilding good governance, which indicates that employees remain critical in combating all forms of unethical behavior (Miceli et al., 2008). Despite the fact that various governments have tried to encourage their employees to be involved in whistleblowing, there is a lack of evidence to support their decision to blow the whistle, and the determinants of whistleblowing in government agencies have not been fully studied. Our current studies provide important evidence of the strong relationships between POP, PSM, PSW, WHE, WHU, and WBI by using a sample of employees who work for government agencies in the US and Indonesia.

Specifically, our main findings can be presented as follows. First, in Study 1, we have identified a positive and significant effect on the relationship between POP and WHU as well as POP and WBI, where POP encourages employees’ intention to blow the whistle. In addition, we also discovered an indirect effect between POP and WBI through WHU. Our findings indicate that POP can be considered a ‘silver bullet’ that triggers employees’ decision to reveal wrongdoing in government agencies. That is, the higher the perceived level of protection, the higher the employee’s intention to engage in whistleblowing. Furthermore, through the understanding of WPA, this may lead them to make the decision to blow the whistle. In this regard, WHU helps in the process of whistleblowing. In the US context, there are laws regarding whistleblower protection that include citizens in general, working in both the private and public sectors (i.e., the Sarbanes–Oxley and Dodd–Frank Acts); this may guide federal agencies in the US to establish organizational protection for whistleblowers. Hence, protection for whistleblowers has become prominent in the US, which may trigger employee intention to report wrongdoing (Brink et al., 2017).

In line with this, the literature shows that an observer will report wrongdoing when he or she feels safe and comfortable to do so (Miceli & Near, 2005). Our findings support previous research conducted by Caillier and Sa (2017), Ugaddan and Park (2019) and Cho and Song (2015), where POP was shown to have a significant positive effect on WBI, mediated by WHU. However, in Study 2, conducted in Indonesia, we did not find similar effects for this emerging economy. We argue that this difference is potentially due to the weakness of WPA in Indonesia. In this context, WPA in Indonesia does not yet exist and there are no laws governing whistleblowing protection as there are in the US, where there is a tradition of public sector whistleblowing protection at federal level. Since there is not enough protection for whistleblowers in general in the Indonesian context, this has implications for the intention of government employees in Indonesia to blow the whistle. They are more likely to remain silent when considering the serious repercussions of blowing the whistle. A study conducted by Latan et al. (2021) in Indonesia found that the perceived seriousness of threats had a negative effect on employee intention to blow the whistle. That is, the seriousness of threats due to lack of protection is a disincentive for whistleblowing.

Second, we found a positive and significant effect on the relationship between PSM and WHU, as well as PSM and WBI, in Study 1 and Study 2, where PSM drives employees to act after observing misconduct. Moreover, we also discovered an indirect effect between PSM and WBI through WHU. PSM generally involves characteristics such as commitment to public interest, upholding social justice, compassion and self-sacrifice (Perry & Wise, 1990; Perry et al., 2010). This motive is commonly found in government agencies and non-profit organizations. In fact, Scheetz and Wilson (2019) report that PSM in non-profit organizations is higher than in for-profit organizations in relation to whistleblowing intention. An employee who has high PSM is usually less motivated by monetary rewards (unlike prosocial behavior), but is motivated by altruistic motivation; that is, to help and sacrifice themselves for the public good. Therefore, the existence of PSM promotes whistleblowing in government agencies. People with high PSM tend to adhere to ethical values and virtues. These people are usually willing to sacrifice themselves to serve the public interest, rather than fulfilling their own personal interests. Therefore, when wrongdoing is related to the public interest and the wellbeing of others, such individuals are likely to blow the whistle. However, this action is not always easy to undertake in the public sector, without having WHU. Meanwhile, PSM will encourage observers to learn the best methods to disclose wrongdoing without suffering retaliation. Several previous studies corroborate our findings (Caillier, 2017b; Cho & Song, 2015; Scheetz & Wilson, 2019), with PSM encouraging civil servants at government agencies to blow the whistle, either directly, or indirectly through WHU.

Third, we found evidence of a positive relationship between PSW and WHU, along with PSW and WBI in both Study 1 and Study 2, where PSW drives the intention to blow the whistle. Also, we found a mediating role played by WHU in the relationship between PSW and WBI. PSW is a consequence of illegal, immoral or illegitimate practices that can affect public trust in general. PSW is often associated with financial and non-financial losses due to misconduct. An observer often considers PSW before deciding to blow the whistle. That is, PSW which has a negative and harmful impact on society will encourage observers to stand up and report it. In addition, a sense of personal responsibility and motivation to serve the public raises the intention of employees to engage in whistleblowing, revealing the seriousness of wrongdoing. Hence, PSW will also influence WHU, and how retaliation and threats as a result of reporting misconduct can be minimized. Generally, anonymous reporting channels are preferred when confronted with the seriousness of wrongdoing. Our findings are in line with what previous studies have reported in relation to the seriousness of wrongdoing (Casal & Bogui, 2008; Keil et al., 2018; Latan et al., 2021; Near & Miceli, 1986), whereby the higher the potential losses caused by wrongdoings, the more likely observers are to engage in whistleblowing.

Fourth, we found evidence of a positive relationship between WHE and WHU, along with WHE and WBI in Study 1 and Study 2, where WHE increases intention to blow the whistle. Also, we found a mediating role played by WHU in the relationship between WHE and WBI. WHE is a form of organizational support, which is intended to help employees when faced with challenging ethical situations. At root, WHE guides employees in how to behave and manage reporting upon observing misconduct in the workplace. WHE aims to answer the following questions: what types of wrongdoing should be reported, how should they be reported, why should they be reported and to whom (Culiberg & Mihelič, 2017; Vandekerckhove & Lewis, 2012). WHE will influence employees’ understanding of whistleblowing when it is implemented in the organization. When employees fully understand whistleblowing, this increases the likelihood of them taking part in it. Several previous studies support our findings (Caillier, 2017a; Cho & Song, 2015; Near & Miceli, 1986), resulting in a positive relationship between WHE and WBI through the mediating influence of WHU.

Finally, we found a positive and significant effect on the relationship between WHU and WBI. In addition, we also found no significant differences between those who have and those who do not have whistleblowing experience in Study 1. Given the understanding of whistleblowers in relation to types of wrongdoing and their knowledge of reporting channels, this is a factor that plays an important role in the whistleblowing process. A person will be more likely to act when he/she has sufficient understanding of this process. An observer who knows what to do after observing misconduct increases the likelihood of him/her becoming involved in whistleblowing. WHU was the main weapon for whistleblowers when deciding to blow the whistle. Our findings fully support the role of WHU in WBI; therefore corroborating previous research that indicates a positive relationship between WHU and WBI (Dungan et al., 2019).

Our studies, therefore, provide the state-of-the-art literature with a unique blend of corroboration and unexpected findings, and trigger a number of theoretical and practical implications for the scientific progress of whistleblowing research, as follows. In terms of theoretical implications, our findings add new evidence and advance insight into whistleblowing in government agencies. More precisely, our studies can be considered one of the first pieces of empirical research to examine the determinants of whistleblowing in government agencies using two field studies. While most prior works have relied on a single study (Mesmer-Magnus & Viswesvaran, 2005; Miceli et al., 2008), such as using a sample collected from a single country or organization, the understanding of whistleblowing across countries is still limited (Vandekerckhove et al., 2014). In addition, our research contributes theoretically to the development of the prosocial organizational behavior model (Dozier & Miceli, 1985) and the social information processing model (Gundlach et al., 2003) by adding new empirical evidence.

In terms of practical implications, our findings offer the following suggestions for government agencies, stakeholders, and related authorities. The sample we analyzed in the US case prefers to report wrongdoing when there is perceived protection from government agencies. Meanwhile, in the case of Indonesia, employees were reluctant to blow the whistle due to the weakness of WPA in their cultural context. We suggest that government agencies in the US continue to increase organizational support for federal employees. Furthermore, government agencies in Indonesia need to develop a more comprehensive organizational protection system. As noted by Vaughn (2012), WPAs do not function well in a number of countries, preventing observers from taking action on wrongdoing and instead encouraging them to choose to remain silent. In addition, investing in WHE would be a better way to allocate resources, rather than investing in monetary rewards to educate employees about how they should behave when faced with challenging ethical situations. Finally, motivation to serve the public needs to be developed early among employees; this has proven to be a dominant factor in combating unethical behavior in government agencies. Government agencies may need to socialize ethical values and virtues among employees, as well as moral standards that apply within the organization.

### Final Remarks, Limitations, and Future Research Avenues

Although these studies make incremental contributions to the whistleblowing literature, we acknowledge several limitations that should be noted. First, with regard to the dataset used in Study 1, we rely solely on data from the 2010 MPS survey conducted by MSPB. Considering that this dataset is archival in nature and that we did not collect it ourselves, as well as involving a very large number of cases, we cannot ensure that the dataset is free from error. Also, we were limited in the nature of the question items that could be adopted from this survey. In addition, the 2010 MPS survey includes a cross-sectional survey design; therefore, we cannot fully claim to preserve causality relationships between variables over time. A study conducted by Near and Miceli (2008) comparing three MSPB surveys from 1980, 1983 and 1992 found differences in coefficients of standardized regression and overall findings. Second, several biases, such as common method bias or social desirability bias, may still interfere with our results, even though we have tried to minimize them. Based on our best knowledge, such biases cannot be completely eliminated when the survey method is based on a self-reporting technique. Finally, our studies are limited with regard to the relationships between variables tested by taking the MSPB survey. There are a number of determinant variables that may not have been included in our model, such as demographic factors (Erkmen et al., 2014), individual characteristics of the whistleblower (Gao & Brink, 2017), situational factors (Cassematis & Wortley, 2013; Culiberg & Mihelič, 2017) and the organizational environment (Mesmer-Magnus & Viswesvaran, 2005; Miceli et al., 2008), all of which might influence whistleblowing intentions in government agencies.

Based on these limitations, we would suggest the following directions for future research. First, future studies may need to consider collecting data over time when testing the relationships between variables in the realm of quantitative research into whistleblowing (Near & Miceli, 2008). We believe that longitudinal or time-series data will work better than a cross-sectional design to ensure consistency of results. As far as we know, there are no existing studies that use longitudinal/time-series designs, other than the MSPB surveys. Therefore, this scope could be expanded to enrich the corpus of whistleblowing research. In addition, future researchers may consider alternative experimental designs to ensure the robustness of the causality relationship in the proposed model. Second, there are a number of research calls related to whistleblowing (Culiberg & Mihelič, 2017; Gao & Brink, 2017; Vandekerckhove & Lewis, 2012) which have not yet been addressed. For example, there is a lack of studies relating to the recipients of whistleblowing reports. How a report is followed up and its effect on misconduct will sway the judgment of other whistleblowers regarding how to behave in future. In addition, the effect of whistleblowing acts on firm performance after the period of reporting has not yet been fully explored. Third, future studies might consider the size of the organization in relation to whistleblowing in government agencies (Brown & Lawrence, 2017). Finally, whistleblowing scholars need to pay attention to conducting research involving case studies from real-world cases. Using qualitative approaches such as interviews with whistleblowers would enable us to more deeply understand the motivations and potential forms of retaliation behind whistleblowing acts.

## Supplementary Information

Below is the link to the electronic supplementary material.

Supplementary file1 (PDF 57 KB)
