# Supplementary material for: What Makes You a Whistleblower? A Multi-Country Field Study on the Determinants of the Intention to Report Wrongdoing
Source: J Bus Ethics. 2022 Mar 25;183(3):885–905. doi: 10.1007/s10551-022-05089-y (PMC8949648; doi:10.1007/s10551-022-05089-y)
Supplement: Supplementary file 1 — Supplementary file1 (PDF 57 KB) [file 10551_2022_5089_MOESM1_ESM.pdf]

Supplementary Materials

Appendix A: Study 1

Sample and Data Collection

We chose to use the MPS data based on two considerations. First, the survey involved a large amount of data from US federal employees related to their intention to blow the whistle, and the breadth of this data is not contained in previous survey-based studies. As highlighted by Near and Miceli (2008), the MPS data has proven very useful, because it gives the possibility of large-scale random sampling that allows us to generalize results across the population, and therefore provides a high level of confidence in our findings. In addition, such large sample sizes are rarely made available for such purposes. Second, previous studies in the whistleblowing arena have used MPS surveys from 1980, 1983 and 1992 (Miceli and Near 1984, 1989, 2002), the results of which have had a real impact on the progress of whistleblowing research.

The MSPB has the statutory responsibility to assess the health of federal merit systems in the US, including to protect the values of integrity, ensure quality public service, efficiency, and fair treatment of employees within US federal agencies. The board was established in 1979 to administer MPS periodically. The 2010 MPS is a US government-wide survey of federal employees that solicits their opinions and experiences related to prohibited personnel practices and whistleblower protection issues. Specifically, topics covered in the 2010 MPS include various issues related to whistleblowing (e.g., PSW, WHE, WHU and WBI), workforce motivation (e.g. PSM) and protection from coercion and reprisals (e.g. POP). The 2010 MPS was administered to permanent, full-time federal employees across the 18 departments and 6 independent agencies. Those departments and agencies accounted for over 97 percent of the permanent, full-time federal workforce. Therefore, the survey results provide a reasonable representation of opinions across US government-wide federal employees.

Given that the sampling frame for the 2010 MPS was known and tracked through records from the Office of Personnel Management's (OPM) central personnel data file, probability sampling was used. Employees were selected through stratified random sampling and the strata (groups surveyed) were designed to provide usable measures of employee opinion. For almost all employees, the survey was administered online through email invitation and a dedicated, secure web site. At the request of the Department of Transportation, MSPB distributed mail surveys to approximately 1,300 employees in the Federal Aviation Administration, who could not receive or respond to an online survey. Employees were informed that survey participation was voluntary and that their responses would be very important to policy makers. In August 2010, 71,970 employees were invited to complete the survey, during which there were two waves of distribution: 1) 70,675 employees were sent a link to the online survey (electronic) through an email solicitation; 2) the remaining 1,295 were contacted via mail survey. Surveys were reviewed for completeness and legibility (for mail surveys). To be accepted, a survey had to contain valid responses.

In order to increase the response rate, MSPB took the following two steps. First, the cover letter sent with the questionnaire emphasized: (a) the strict confidentiality of responses (i.e., no data would be disclosed to anyone that could be used to identify individual participants); (b) the importance of the responses (i.e., that responses would help policy makers understand how to improve the federal workplace and possibly legal policy concerning whistleblowing). Second, a follow-up postcard was mailed about three days after the first mailing. The postcard was intended to serve as a reminder; additionally, several hundred people who received postcards called the MSPB to say they had not received questionnaires, which were then mailed to them. The time period provided by MSPB for completing the survey was 5 days after the email/mail was received.

Instruments

The questionnaire used in the 2010 MPS survey was designed by MSPB. The items used in the 2010 MPS survey were mostly taken from previous surveys from 1980, 1983 and 1992 and have been used in several empirical studies (Caillier 2017b; Cho and Song 2015; Dungan et al. 2019; Miceli and Near 2002, 1985). Hence, the validity and reliability of the items have been proven and are trusted. In the 2010 MPS survey, there were about 325 items used to assess prohibited personnel practices, whistleblower protection issues and other personal and organizational factors (the full questionnaire is accessible at https://www.mspb.gov/studies/surveys.htm).

Appendix B: Study 2

Sample and Data Collection

Specifically, we used target respondents with a similar profile to those in Study 1. We used employees who work in government agencies in Indonesia to answer these questions, through a non-probability sampling technique which is considered precise, in order to reduce time and costs. We entered items from the 2010 MPS survey into the questionnaire link and sent them to these respondents via email. Previously, we asked permission from each agency to take part in our survey. We received responses from around 16 agencies that were willing to participate in the survey.

We initially set aside 5 months for the data collection process, which took place between October 2019 – February 2020. We used electronic surveys and successfully sent 532 emails. We obtained the email addresses of each employee through a database provided by personnel management. In order to increase the response rate, a reminder e-mail was sent at the end of each month. Given the Covid-19 outbreak that occurred in December 2019, we sped up the data collection process, ending it in January 2020. In addition, employees were assured of their anonymity and that their names and details would not be disclosed. Informed consent was considered to have been obtained when employees completed the survey and sent it back to us, wherein they agreed that their responses would be used in this study. We considered two waves of responses for the purpose of non-response bias testing; i.e., those who responded before and after the deadline.

Instruments

The scales and measurement items used in Study 2 were taken from the 2010 MPS survey. Similar to Study 1, these items were chosen because they have gone through a series of tests for scale development and have been previously tested.

References

Caillier, J. G. (2017b). Public service motivation and decisions to report wrongdoing in U.S. federal agencies: Is this relationship mediated by the seriousness of the wrongdoing. The American Review of Public Administration, 47(7), 810–825.

Cho, Y. J., & Song, H. J. (2015). Determinants of whistleblowing within government agencies. Public Personnel Management, 44(4), 450–472.

Dungan, J. A., Young, L., & Waytz, A. (2019). The power of moral concerns in predicting whistleblowing decisions. Journal of Experimental Social Psychology, 85, 103848.

Miceli, M. P., & Near, J. P. (1984). The Relationships among Beliefs, Organizational Position, and Whistle-Blowing Status: A Discriminant Analysis. Academy of Management Journal, 27(4), 687–705.

Miceli, M. P., & Near, J. P. (1985). Characteristics of organizational climate and perceived wrongdoing associated with whistleblowing decisions. Personnel Psychology, 38(3), 525–544.

Miceli, M. P., & Near, J. P. (1989). The incidence of wrongdoing, whistle-blowing, and retaliation: Results of a naturally occurring field experiment. Employee Responsibilities and Rights Journal, 2(2), 91–108.

Miceli, M. P., & Near, J. P. (2002). What makes whistle-blowers effective? Three field studies. Human Relations, 54(4), 455–479.

Near, J. P., & Miceli, M. P. (2008). Wrongdoing, whistle-blowing, and retaliation in the U.S. government: What have researchers learned from the merit systems protection board (MSPB) survey results? *Review of Public Personnel Administration, 28*(3), 263–281.
